# Midwives as agents of change: a qualitative analysis of midwives’ experiences with abortion care provision in Canada

**DOI:** 10.1080/26410397.2025.2548657

**Published:** 2025-08-18

**Authors:** CA Mattison, V Perrault, A Hibbert, F Pittson, J Robinson

**Affiliations:** a Research Affiliate, Department of Women's and Children's Health, Karolinska Institutet, Stockholm, Sweden; Director, Insight Research & Policy Inc., Guelph, Canada. *Correspondence*: cristina.mattison@ki.se; b Midwifery Technical Expert, Canadian Association of Midwives, Montreal, Canada; c Coordinator, Association Strengthening, Canadian Association of Midwives, Montreal, Canada; d Project Coordinator, National Programs, Canadian Association of Midwives, Montreal, Canada; e Program Development & Research, Canadian Association of Midwives, Montreal, Canada

**Keywords:** midwifery, midwives, abortion, reproductive justice, sexual and reproductive health and rights

## Abstract

Midwives possess the skills and competencies required to provide abortion care in Canada, yet their role is constrained in health systems. They are well suited to address barriers to abortion access related to geographical and social inequities, which deprive many Canadians of essential healthcare and impede reproductive justice. To address current gaps, this study explores midwives’ experiences providing abortion care in Canada. Qualitative data were collected from 25 in-depth interviews and three focus group discussions with midwives between August and December 2023. Using reflexive thematic analysis, we explored how midwives work in communities to provide or work toward providing abortion care, including health system facilitators, barriers, and their values, needs, and preferences for implementation. Findings highlight the barriers midwives face, including regulatory restrictions and a lack of flexible funding arrangements. Despite these challenges, midwives are leveraging their skills to advance reproductive justice, offering culturally safe, client-centred abortion care to underserved populations, including uninsured individuals. The study also identifies facilitators, such as applying midwifery values and philosophies to provide the midwifery model of abortion care. This research contributes to the growing body of knowledge on midwifery and abortion care, advocating for the removal of regulatory and funding barriers that limit midwives’ potential to provide comprehensive sexual and reproductive healthcare. The findings have significant implications for policymakers and health system leaders in Canada and beyond, calling for the optimisation of midwives’ roles to improve access to abortion care and advance reproductive rights globally.

## Plain language summary

Midwives in Canada have the skills and knowledge to provide abortion care, but they face challenges in doing so within health systems. This study looks at the experiences of midwives across the country who are providing or working towards providing abortion care. The research involved 24 interviews and 3 focus groups with midwives, aiming to understand the barriers they face and what helps them offer this care. Many midwives encounter obstacles like strict regulations and a lack of flexible funding, which limit their ability to provide abortion care. Despite these challenges, midwives are using their skills to help ensure everyone can access safe, respectful, and client-centred abortion care, especially in underserved communities. This includes offering care to people who are uninsured or face difficulties in accessing healthcare. The study also found that midwives’ values and approaches to care, such as their focus on informed choice and continuity of care, help them deliver high-quality abortion services. The findings also suggest that changes are needed in policies and funding to remove barriers that prevent midwives from providing comprehensive care. These changes would improve access to abortion care and advance reproductive rights, not just in Canada but globally.

## Résumé

Les sages-femmes possèdent les aptitudes et les compétences requises pour prodiguer des soins d’avortement au Canada, mais leur rôle est limité dans les systèmes de santé. Elles sont bien placées pour lever les obstacles à l’accès à l’avortement liés aux inégalités géographiques et sociales, qui privent beaucoup de Canadiens des soins de santé essentiels et entravent la justice reproductive. Afin de combler les lacunes actuelles, cette étude explore l’expérience des sages-femmes dans la prestation de soins d’avortement au Canada. Des données qualitatives ont été recueillies à partir de 25 entretiens approfondis et de trois discussions par groupes thématiques avec des sages-femmes entre août et décembre 2023. À l’aide d’une analyse thématique réflexive, nous avons examiné la manière dont les sages-femmes travaillent dans les communautés pour assurer ou s’efforcer de prodiguer des soins d’avortement, y compris les facilitateurs du système de santé, les obstacles ainsi que leurs valeurs, leurs besoins et leurs préférences en matière de mise en œuvre. Les résultats mettent en évidence les obstacles que rencontrent les sages-femmes, notamment les restrictions réglementaires et le manque de flexibilité des modalités de financement. En dépit de ces difficultés, les sages-femmes mettent à profit leurs compétences pour faire progresser la justice reproductive, en offrant des soins d’avortement culturellement sûrs et centrés sur la cliente à des populations sous-desservies, y compris les personnes non assurées. L’étude identifie aussi les facteurs facilitants, comme l’application des valeurs et des philosophies de la profession de sage-femme à la prestation du modèle de soins d’avortement. Cette recherche contribue à la somme croissante de connaissances sur le métier de sage-femme et les soins d’avortement, en préconisant la levée des obstacles réglementaires et financiers qui limitent la capacité des sages-femmes à assurer des soins de santé sexuelle et reproductive complets. Les conclusions ont des conséquences importantes pour les décideurs et les responsables des systèmes de santé au Canada et ailleurs, appelant à l’optimisation du rôle des sages-femmes afin d’élargir l’accès aux soins d’avortement et de faire progresser les droits reproductifs dans le monde.

## Resumen

Las parteras poseen las habilidades y competencias necesarias para proporcionar servicios de aborto en Canadá; sin embargo, su rol está limitado en los sistemas de salud. Están bien preparadas para abordar las barreras al acceso al aborto relacionadas con inequidades geográficas y sociales, que privan a muchas personas canadienses de servicios de salud esenciales e impiden la justicia reproductiva. A fin de abordar las brechas actuales, este estudio examina las experiencias de parteras con la prestación de servicios de aborto en Canadá. Se recolectaron datos cualitativos de 25 entrevistas a profundidad y tres discusiones en grupos focales con parteras entre agosto y diciembre de 2023. Utilizando análisis temático reflexivo, examinamos cómo las parteras trabajan en comunidades para proporcionar o trabajar hacia proporcionar servicios de aborto, así como los facilitadores y las barreras de los sistemas de salud, y sus valores, necesidades y preferencias de implementación. Los hallazgos destacan las barreras enfrentadas por las parteras, tales como restricciones normativas y la falta de acuerdos de financiación flexibles. A pesar de estos retos, las parteras aprovechan sus habilidades para lograr avances en la justicia reproductiva, ofreciendo servicios de aborto centrados en las usuarias y culturalmente seguros a poblaciones desatendidas, incluidas las personas sin seguro. Además, el estudio identifica los facilitadores, tales como aplicar los valores y las filosofías de las parteras para ofrecer el modelo de partería en la atención al aborto. Esta investigación contribuye al creciente conjunto de conocimientos sobre partería y servicios de aborto, y aboga por la eliminación de barreras normativas y de financiación que limitan el potencial de las parteras para proporcionar servicios de atención integral a la salud sexual y reproductiva. Los hallazgos tienen implicaciones significativas para formuladores de políticas y líderes de los sistemas de salud en Canadá y más allá, e instan a optimizar los roles de las parteras para mejorar el acceso a los servicios de aborto y promover los derechos reproductivos a nivel mundial.

## Introduction

Barriers to abortion access deprive a significant number of Canadians from accessing their rights as defined under the Canada Health Act.^1,2^ These barriers, related to geographical and social inequities, impede reproductive justice in Canada. Abortion care in Canada is not evenly distributed, with services concentrated in major urban centres and along the border with the United States (US).^2^ Canada’s vast geography is bordered by three oceans (Arctic, Pacific, and Atlantic), spans six time zones, and has eight distinct climate regions, which adds to the complexity of healthcare delivery.^3^ In some provinces and territories, services are only available for up to 13 weeks gestation.^2,4–6^ This results in many travelling outside their region for many hours by road or plane, taking additional time off work, arranging care for children, facing negative judgment and discrimination and significant financial barriers to access the healthcare they need.^4,6^ For example, Indigenous, Black, and People of Colour (IBPOC) and 2SLGBTQIA+ people are disproportionately impacted, experiencing abortion access barriers that include logistical barriers, mistreatment by health professionals in medical settings, stigma at societal and community levels, and lack of supportive follow-up care.^7,8^ Despite abortion care coverage for citizens and permanent residents under provincial, territorial, and federal health insurance systems,^1^ there are many people for whom costs are not covered (e.g. international students, migrants, and undocumented people).^9,10^

In this article, we recognise abortion as a normal and routine part of pregnancy care.^11^ Aligning with the World Health Organization’s guidance, this includes care related to early pregnancy loss (miscarriage, spontaneous abortion, and missed abortion), induced abortion (medication or procedural interruption of pregnancy), incomplete abortion (incomplete passage of the products of conception), and fetal death.^11,12^ Comprehensive abortion care includes information and counselling, abortion management, and post-abortion care. It is part of the care midwives provide globally, as affirmed by the International Confederation of Midwives.^12–14^ The Canadian Midwifery Regulators Council’s Canadian Competencies for Midwives also states that midwives offer abortion counselling and provision, recognising that regulations differ by jurisdiction.^15^ Midwifery is a publicly funded and regulated health profession in all Canadian provinces and territories. However, there is significant variation in midwives’ ability to provide contraceptive and abortion care across jurisdictions. Currently, midwives can prescribe and manage medical abortion under their own authority in only two provinces (Quebec and Saskatchewan). Four provinces and territories (Alberta, Ontario, Northwest Territories, and Nunavut) maintain restrictive policies requiring physician oversight, and the remainder have regulatory barriers that prevent midwives from providing abortion care altogether.

Midwives possess the skills, knowledge, and competencies required to provide abortion care in Canada, yet their role is constrained in health systems. Regulatory barriers, including prescribing authority for midwives and funding barriers, currently limit midwives’ ability to provide abortion care services.^16^ The Canadian midwifery model of care,^17^ scope of practice, and training are unique among other health professions, and this approach and philosophy make midwives an ideal health profession to support equitable access to abortion care.^13^ As highlighted by Action Canada for Sexual Health and Rights in a recent policy brief on increasing abortion access through midwife-led care, midwives are ideal providers as they: (1) currently manage uncomplicated pregnancy loss, which is a related clinical skillset to managing medical abortion, (2) collaborate with other health professionals to provide client-centred care, (3) provide quality pre- and post-abortion counselling; and (4) understand that abortion care fits within the midwifery philosophy, which includes continuity of care, informed choice, and providing care which is client-centred.^18^ Midwives also provide supportive care in complex abortion situations, including support for clients with medical mistrust.

This research study is part of a larger project led by the Canadian Association of Midwives within the University of British Columbia’s “CART Access project: Advancing access to abortion for underserved populations through tools for healthcare professionals and people seeking care”.^19^ Within the project, the Canadian Association of Midwives has developed evidence-informed advocacy tools for midwifery associations, midwives, regulators, midwifery education programmes, and collaborating health professionals, including a *National Strategy for Midwife-led Abortion Care in Canada*, briefing notes, an advocacy road map, and a stakeholder mapping tool.^20^

As noted above, health systems in Canada are not meeting the sexual and reproductive care needs of women, trans, and nonbinary people. Many continue to face significant barriers to culturally safe, appropriate, and timely access to abortion care.^6^ Enhancing how abortion care is delivered is vital to supporting equitable access to sexual and reproductive healthcare in Canada. To address current gaps in care, this study explores midwives’ experiences with providing abortion care in Canada. Specifically, we interviewed midwives working in communities across Canada to understand why they provide abortion care or why they are working to provide this type of care. We also asked midwives to share how they have integrated abortion care into their practice, as well as what facilitators and barriers they encountered.

### Reproductive justice

The reproductive justice framework underpinned our approach to the research, analysis, and recommendations.^21,22^ We recognise that before moving towards reproductive justice, we must first recognise the past and current abuses of women, trans, and nonbinary peoples’ reproductive bodies.^22^ Reproductive oppression in Canada includes a range of intersecting oppressions that perpetuate systemic inequalities and injustices. These include but are not limited to the enduring legacies of racism and colonisation, manifested through medical colonialism, obstetric violence, and anti-Indigenous racism, which not only result in the denial of healthcare and violence towards Indigenous communities but also in structural policies that strip away reproductive care and birthing resources from these communities.^16,23,24^ Moreover, forced and coerced sterilisation policies target First Nations, Inuit, Métis, and Black individuals and those at the intersections of gender, race, poverty, and disability.^25,26^ The imposition of contraception without consent further violates bodily autonomy, and coerced abortions, a form of reproductive control, are perpetuated through oppressive laws and policies.

Systemic discrimination and structural barriers continue to limit access to inclusive reproductive care, specifically safe abortion services, for 2SLGBTQI+ people.^25^ There are also systemic biases in family policing, particularly affecting IBPOC communities, which are reflected in the disproportionate rates of child and newborn apprehensions within these communities.^27,28^ A lack of access to safe and sustainable communities is exacerbated by housing crises, food insecurity, police brutality, and lack of access to safe drinking water.^29^ Individuals with disabilities encounter numerous barriers to accessing comprehensive reproductive care, including inaccessible facilities and transportation. Lastly, the lack of healthcare coverage for uninsured groups, such as undocumented people, migrant workers, and international students, creates significant financial barriers to accessing essential healthcare services.^4^

Reproductive justice is a social movement and intersectional contemporary framework for activism, which builds on and bridges the advocacy work of women of colour and grassroots health organisations in the United States.^30^ SisterSong Women of Color Reproductive Justice Collective is a Southern network in the United States focused on addressing the policies and systems that impact the reproductive lives of those facing systemic and routine barriers to care.^30^ The movement recognises that inequitable access to abortion care is but one manifestation of healthcare inequities. Reproductive justice work includes affirming rights to care and calls to action towards improved access for all to comprehensive sexual and reproductive care, which includes contraception, comprehensive sex education, options counselling, recognising and responding to family violence, and perinatal care.^30^ There are four primary principles of reproductive justice:

*“The human right to own our bodies and control our future. The human right to have children. The human right to not have children. The human right to parent the children we have in safe and sustainable communities.”*^31^

## Methods

We undertook an exploratory qualitative study that used semi-structured interviews and focus group discussions with midwives to gain insights into their experiences with abortion care provision in Canada. The data generated were analysed deductively using Braun and Clarke’s reflexive thematic analysis.^32^ The analysis was shaped by an existing theoretical framework, reproductive justice, which provided a “lens” through which to generate themes from the data. Using a descriptive and exploratory orientation, codes emerged from the data and were not predetermined.

### Data collection

Data collection consisted of semi-structured research interviews and focus group discussions. Both approaches were guided by the same interview questions and were designed to explore participants’ experiences with midwife-led abortion care. However, they differed in recruitment strategy. A multi-stage purposive sampling approach was used to identify potential participants. The first stage consisted of a sample of midwives identified by the research team and the Steering Committee to participate in the semi-structured research interviews. The second stage was driven by respondents and consisted of purposive sampling by asking research participants to identify additional potential participants. The third stage included sharing the information letter and consent form through midwifery and abortion provider networks to participate in focus group discussions. These networks included the Canadian Association of Midwives’ weekly newsletter for members and the National Abortion Federation Canada’s outreach to midwives who completed their medical abortion training. Research interviews were conducted online (Microsoft Teams or Zoom) or in person. All focus group discussions were conducted online via Zoom.

Inclusion criteria were participants over the age of 18, French or English speakers, and midwives either currently providing abortion care or in the process of implementing abortion services. The interview guide was an iterative process informed by themes as they emerged from the initial stages of data collection and analysis. We asked each midwife to share their thoughts on midwife-led abortion care and to share stories on why they provide abortion care or stories related to why they are working to provide this type of care. We also asked midwives to share how they have integrated abortion care into their practice, as well as what facilitators and barriers they encountered. As data collection progressed, the guide was refined based on emerging insights. These refinements involved adding targeted prompts to explore gaps in understanding, primarily processes for navigating and addressing barriers in health system arrangements, such as generating medical directives, interprofessional collaboration, and remuneration. Written informed consent was obtained from each participant. The interviewers (CM, VP) audio-recorded the interviews and focus groups and took field notes. The program Otter.ai was used to transcribe the audio files. Transcriptions were reviewed (CM) for accuracy and the removal of identifiers. Data were collected until saturation or when insights drawn from the analysis stages became exhausted. The research team held weekly meetings to discuss data collection and assess the emergence of new themes, and once sufficient depth and consistency were achieved, data collection concluded. The approach to saturation was guided by Braun and Clarke’s reflexive thematic analysis, which is discussed in greater detail below.^32^

A steering committee for the project was created to advise and provide guidance to the team. The steering committee met regularly and provided critical feedback on drafts of the national strategy and policy resources. The committee included representation from various stakeholders and midwifery associations, including the National Council of Indigenous Midwives, the Canadian Alliance of Racialised Midwives, the Canadian Caucus of Queer and Trans Midwives, Canadian Midwifery Regulators Council, Action Canada for Sexual Health and Rights, and the National Abortion Federation Canada.

### Data analysis

Data analysis occurred in English and French (CM, VP, AH, FP), and Braun and Clarke’s reflexive thematic analysis was applied, facilitating a systematic and in-depth examination of the data. The approach allowed for identifying and categorising recurring patterns within the data.^32^ Broadly, the approach has six parts: (1) getting to know the data; (2) coding the data; (3) creating themes from the coded data; (4) reviewing the themes; (5) refining, defining, and naming themes; and (6) writing up.^32^

We analysed the data using the qualitative software ATLAS.ti. We approached the data using a reproductive justice and intersectional lens to critically consider participating midwives’ diverse perspectives, values, needs, and preferences. Our analysis was shaped by an understanding of how reproductive oppression operates in Canada – through the historical and ongoing impacts of racism, colonialism, coerced sterilisation and contraception, systemic ableism, anti-2SLGBTQI+ discrimination, and barriers faced by uninsured people.^20,31^ We were especially attentive to how participants’ multiple and overlapping identities intersect with their practice contexts and the communities they work in. Particular consideration was given to interviewing midwives working in northern and remote communities and those serving clients who are uninsured, including undocumented people, migrant workers, and international students. These insights guided our analysis and interpretation of midwives’ experiences with abortion care within broader systems of reproductive and social injustice. We were mindful of protecting participant confidentiality and limited the demographic details provided in the results section.

We also incorporated strategies that increased interaction and member checking with participants and local stakeholders throughout the research process. First, we used the Canadian Association of Midwives’ annual conference in October of 2023 to host a guided discussion with members. We presented initial findings and held breakout groups with midwives to ensure that our findings were contextually appropriate and reflected midwives’ experiences across Canada. Over 90 members participated in the session. We also returned to participants to share results and check for accuracy and resonance with their lived experiences. The Steering Committee reviewed and provided feedback throughout the project.

### Researcher reflexivity

Throughout the research process, the project team engaged in reflexivity both individually and collectively, actively considering our positionalities and how these may have shaped the study’s design, recruitment strategy, interviews, and analysis. We are a multinational, multilingual team based in Quebec and Ontario, Canada, bringing a range of experiences related to abortion care, including as health professionals, programme implementers, advocates, applied researchers, and individuals with lived experience accessing abortion services.

CM holds a PhD and brings over 20 years of experience in health systems and policy research and consulting, specialising in sexual and reproductive health and rights. The majority of the team was affiliated with the Canadian Association of Midwives: VP (Midwifery Technical Expert), JR (Global Manager, Projects and Partnerships), AH (Association Strengthening Lead, National Programs), and FP (Project Coordinator, National Programs).

Reflexivity was embedded throughout the project, including through weekly team discussions and individual reflexivity journalling by CM. These practices supported our critical examination of how our values, identities, and assumptions influenced the research. As a team committed to reproductive justice, we continuously reflected on whose voices were missing, the power dynamics inherent in our work, and how to protect the anonymity and confidentiality of participants – particularly given the small number of providers and advocates engaged in this field.

### Ethical approval

On August 21, 2023, the Community Research Ethics Board, located in Waterloo, Ontario, Canada, approved ethics (Research Project #316). Each participant provided written informed consent.

## Results

In the fall of 2023, we conducted 25 research interviews with midwives and three focus group discussions (FGD). The interviews ranged from 35 minutes to 2.5 hours, averaging 55 minutes. We captured insights from midwives representing ten of the 13 provinces and territories. These midwives worked in many communities, including urban, rural and remote, and Indigenous communities. The midwives supported underserved clients with diverse needs (e.g. new immigrants, uninsured people, international students, people who are using drugs, people who are homeless or under-housed, people with low income, queer and trans people, and young people). The midwives who participated in the interviews included racialised midwives, 2SLGBTQI+ midwives, midwives with disabilities, midwives who are immigrants to Canada, and internationally educated midwives.

**Figure 1.**
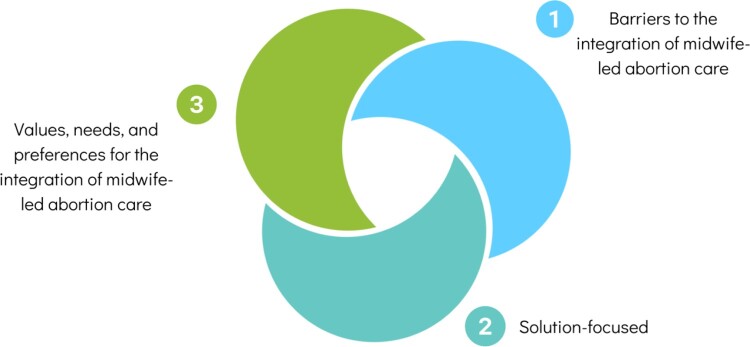
Theme summary figure.

Three main themes emerged in the analysis (Figure 1). The first theme that emerged was challenges around midwives’ integration of abortion care into their practice. Second, midwives described the range of ways they are responding to community needs to provide abortion care. Lastly, midwives discussed their values, needs, and preferences for the integration of midwife-led abortion care into practice. Below, we present each theme and its sub-themes and include illustrative quotations.

### Barriers to the integration of midwife-led abortion care

Regulatory barriers, lack of flexible funding arrangements, and restrictions on how midwives deliver comprehensive sexual and reproductive health services were the main challenges to the integration of midwife-led abortion care raised by participants. Barriers related to the anti-choice movement, both within midwifery and societally, also emerged in the data.

Within the sub-theme of regulatory barriers for midwives and abortion care, participants outlined challenges with legislation. Specifically, participants cited the interpretation of midwifery Acts within jurisdictions and the confusion this creates. Participants described the challenges with interpreting scope and what is included in midwives caring for “normal” pregnancy. Participants felt strongly that abortion was a routine and normal part of pregnancy care.

*“That’s an angle and an interpretation of the Act to do it that way. Right? But it’s also an accurate one. So, like, I don't know, I’m not a lawyer if I'm incorrect about this, but I truly think that it would be very difficult to argue that uncomplicated medical abortion is not part of the scope of practice of midwives.”* [Interview 115]

Some jurisdictions have extra challenges given the regulatory structures that have been set up with respect to midwifery legislation.^33^ In July 2022, the Ordre des sages-femmes du Québec (midwifery regulator) issued a position statement affirming that abortion care, including medical abortion, was part of the midwifery scope. Yet, some opposition to this interpretation remains despite strong political will and inter-regulator support.

*“I feel responsible for this as a midwife. Because the fact that I accompany pregnancies for me, it goes without saying that I should (provide) abortion because it's part of the choices that can be offered when you become pregnant, you know. To me, it just makes sense. I mean, it's really obvious; it's just the way it is. I find it strange that care that's easy to administer, easy to regulate if you like. You know, it's not demanding, you know. I mean, to offer this care, medication abortion.”* [Quote translated from French – Interview 121]

The lack of flexible funding arrangements for midwives to provide comprehensive sexual and reproductive health services was also raised as a key barrier by participants. These challenges were described as payment mechanisms that do not account for midwives’ expertise in care. Participants specifically cited an overall lack of billing mechanisms and restrictive billing schedules that do not allow midwives to bill for the care they provide in the first trimester. In Ontario, the province with the largest midwifery workforce in Canada, most midwives work within a community-based “course of care” model. These midwives are self-employed, working as independent contractors in midwifery practice groups, and payment is made through a billable course of care system. One participant described the system's impacts:

*“What? Do you do any volunteer work? Yeah, I manage miscarriages and terminations. And I have trouble thinking of any other healthcare provider in the system that provides any healthcare for zero compensation. But that is what was expected of us and to see that client through. And sometimes you would get further involved, and they might be home with no one to call in. They're hemorrhaging or looking for emotional support. So as a midwife, this could involve many visits and much of your time, but we've not been compensated.”* [FGD 2]

*“I was hoping to work with them doing IUD insertions and terminations, […] And they were thrilled to have me on, but there was no way to bill, and that was what held it back. It wasn't even the funding; they had funding. And, they had a doc who was willing to do a medical directive, but the fact that we had no billing mechanism for this, and I was okay to volunteer. I actually offered to volunteer one day a week to go down, and I know they needed folks, and we need it, you know, and I was happy to do it. Anyway, they ended up getting funding and through the nurse practitioner who came in to do it instead. So it's about funding, and that's the part that is so frustrating. And our funding model that is so limiting.”* [FGD 3]

Within barriers to the integration of midwife-led abortion care, participants recognised the tension of working within medical directive arrangements and restrictions to the extent to which they can provide abortion care. While medical directives were recognised as a critically important response to current restrictions on midwife-led abortion care, they were also seen as a temporary “patch” and as a mechanism that was tenuous and vulnerable to being removed.

*“When you're working under a medical directive, there are these constraints in place. It's taking the primary care piece out of it in a way because you're still working within a directive model or approach, and so it's like devaluing midwifery as a primary care health professional.”* [Interview 112]

*“[on medical directives] It's really insulting, it's a lot of hoops. […] It should be my OB colleagues at the hospital that are maximising my scope of practice because then it's less for them to do. But that's just not how it works. […] It does leave me very vulnerable to the one person, like let's say she leaves or she moves or whatever, then I start this all over with someone else, and then I have to convince them.”* [ID 124]

Limitations to medical directives and policies related to the conditions for the delivery of medical abortions were raised, particularly in relation to healthcare in rural and remote communities. For example, several participants discussed the crucial role of manual vacuum aspirations (MVA) within abortion care and the need for midwives to have these skills to support the communities in which they work.

*“One of the issues we had as the medical team, and one of the rules, is if […] you're going to do a medical abortion, you need to have a professional in town who can do the emergency aspiration. And that's a problem because they screwed up this summer. And nobody was there for weeks. And it was heartbreaking. […] That was a huge problem. And they're refused. The doctors refused to prescribe medical abortion to women because they didn't have anyone who could do the crash. We were very mad. And we want the midwifery team to be able to step in.”* [Interview 118]

Lastly, the impacts of anti-choice midwives and societal anti-choice values more broadly were cited as barriers to the integration of midwife-led abortion care. Participants discussed barriers within midwifery education and practice settings related to learning from or working with midwives who were anti-choice. *“One of the parts that was a little tricky for me working there was that the head midwife was very anti-abortion. So, we also have to work around that piece as well.”* [Interview 116]

Participants shared concerns with the growth and strength of the anti-choice movement, especially the influence of the post-Roe context in the United States. A few participants also discussed challenges within the province of Alberta, specifically, and the province’s relationship with conservative values.

*“I would say that conservativism here in Alberta does pretty strongly align with the religious base and anti-choice philosophies.”* [Interview 120]

*“I know people are worried about their safety, especially with how our Alberta politics are going.”* [Interview 125]

### Solutions-focused

Midwives described a range of ways in which they were providing abortion care in their community. The sub-themes that emerged focused on the health system arrangements that midwives navigated and put in place to be able to provide care, the midwifery model of abortion care, and how midwives applied the midwifery model of abortion care to provide services for underserved people and address equity in healthcare.

Within health system arrangements, we learned about the conditions required for midwives to deliver abortion care services within health systems that currently have regulatory barriers to midwife-led abortion care. Participants often described the process as identifying barriers and finding solutions to work around the barriers. Medical directives, or standing orders, advanced practice, and advanced authorisation, depending on the jurisdiction, emerged as an essential mechanism for abortion care provision by midwives. Directives are authorising mechanisms that enable the performance of a procedure by another cadre of health professionals. In this case, most often, midwives had medical directives with physicians for *Mifegymiso* for medical abortion.

Flexible funding models, such as Expanded Midwifery Care Models in Ontario, coupled with responsive institutional arrangements, emerged as a strong facilitator for the implementation of midwife-led abortion care. Within this sub-theme, participants also described how identifying barriers and then finding ways to work around these were key:

*“So we just decided we identified it as a barrier and a problem, but we also identified that employment, a salaried model, was what we wanted and was ideal to meet the needs of the midwives and the clients that we wanted to build this program around. And so we simply chose, in a very conscious way, to ignore that barrier and moved ahead with the model that we wanted, in the shape that we wanted it.”* [Interview 105]

Strong interprofessional collaboration enhanced midwives’ roles in providing abortion care. Participants reported a cascading effect, where midwives were recognised for their clinical expertise and were changemakers within comprehensive sexual and reproductive care more broadly. *“We've been able to move things. You know, we're acknowledged for our expertise now”* [Interview 106]. Participants described the process of building strong interprofessional collaborative networks as one that took a lot of time and dedication.

*“We were not welcomed into a lot of these spaces. It takes time. And it takes persistence and, like in a lot of these spaces that we do sit, we are the only midwives, and that's okay, you know, in time that will shift, but at least we have a voice at a lot of tables that we never once did.”* [Interview 108]

Participants highlighted the unique approach of midwives, supported by midwifery values and philosophies. They labelled this a “midwifery model of abortion care”. The model was described as focusing on holistic care across the lifespan, supporting the principles of reproductive justice, and focusing on equity-driven, culturally safe, and community-based abortion care environments. *“It's this person's choice, this person's autonomy and it really has nothing to do with me. My job is actually to find the connections and build the frameworks in the system”* [Interview 125]. One participant described their clinic’s open approach and unbiased counselling when meeting clients as follows:

*“When my clients come, we say, what kind of care are you looking for? Abortion, pregnancy options counselling, or pregnancy care? So that's the first question they get asked, and that tells them from the beginning. Oh, I have options, and there's someone who can answer my questions. No matter what I choose. And no matter what you choose, I will likely be your care provider.”* [Interview 105]

Another participant described in practical detail how providing medical abortion services under the midwifery model of abortion care differs from the currently dominant (physician-led) approaches to abortion care in Canada.

*“We call them two to four hours after they take the medication. And so, as the midwife, sometimes that means a 10 PM call. The midwife on call makes that call. It's in our schedule. It's part of our on-call work, and we do a phone triage visit with the person while they're experiencing the heaviest bleeding. And then we hang up that call and we're still available at 3 AM if they need to call back, but actually they don't. That phone call during that most intense part provides so much reassurance and clarity, and you know, is this normal or not?”* [Interview 105]

Lastly, within the solutions-focused theme, midwives discussed the different ways that they apply the midwifery model of abortion care to meet the needs of underserved groups and address issues of equity in healthcare more broadly. Participants expressed a deep knowledge of and caring for the specific sexual and reproductive health needs of their community. They gave applied examples of intersectional approaches to meeting reproductive health needs. These examples included keeping a stock of *Mifegymiso* in the clinic or funding arrangements with pharmacies for uninsured people, free abortion care supply kits for clients (thermometer, pain and nausea products, informational printouts, pregnancy tests, condoms, and menstrual supplies), the inclusion of interpreters, encouraging the accompaniment of a support person even during COVID-19 restrictions, and offering a safe place within the clinic to have the medical abortion. Participants also described navigating complex health system arrangements most often related to the vast geography of Canada and the lack of available health professionals to provide abortion care, especially with respect to second and third-trimester abortions.

*“The gestational age limit provides a lot of limits to people who may have more complex logistics and might delay beyond their gestational age, just because either they maybe live out of town on a reserve […] All of the complexities of people's lives in the North, and probably in lots of other places as well. But there can be definite barriers. And the limited numbers of spots for surgical abortions.”* [Interview 109]

*“Timing is critical. […] The regional program does abortions one day a week, so they fly in on a Tuesday, they have ultrasound and counselling on Wednesday. And then if they're having a surgical procedure, would be on Thursday. And then if everything goes well, they come home on Friday. It's pretty quick turnaround. I don't love the idea of people flying when they are post anything.”* [Interview 113]

### Values, needs, and preferences for the integration of midwife-led abortion care

Participants described a range of midwife-led abortion care integration considerations. Participants emphasised that abortion care is a part of midwifery care and shared preferences related to midwives’ role as providers of comprehensive sexual and reproductive health and how integration considerations impact the sustainability of the profession.

An overarching theme across interviews and focus group discussions was the recognition that abortion care is part of the care midwives provide: *“There’s this wave of change that's been coming this year, and that continues to allow midwives to step into this”* [Interview 101]. Participants shared the ways in which they would like to see midwifery regulation, scope-of-practice, pharmacopeia, and training better reflect midwives’ role in the provision of comprehensive sexual and reproductive healthcare.

*“I would love to see midwives’ scope changed, you know, from across Turtle Island, so that we could be providers if we wished. That would be the best. I'd also love to be able to provide sexual and reproductive health care across the lifespan.”* [Interview 112]

*“For abortion care, I would also like that midwives do not need to ask for permission to prescribe medication, that it’s basic to treat our clients. And then I will dream of a curriculum that adds that piece and education of future midwives.”* [Interview 103]

Within training and education, participants highlight the importance of practising and maintaining abortion-related skills (e.g. provision of ultrasounds), as well as gaining the necessary skills to perform MVAs. *“I think that a lot of midwives don't have a lot of experience in terms of procedures within the uterus. So again, I think that with additional training or as a like add-on, I would hope to see midwives within that space”* [Interview 117]

*“I would like for the midwives to have a MVA training, just like we have NRP [Neonatal Resuscitation Program], and to be able to redo it, to have resources, to have material and to be able to do like team practices and to recertify every X because, you know, like any skill and we're not going to be doing it every day. What I would love is to have an abortion skills workshop. That's what I want. And I want everybody to be able to, like, access this information and get good at it and keep the skills fresh, just like NRP, just like a standard.”* [Interview 118]

In terms of how comprehensive sexual and reproductive healthcare is delivered by midwives, participants emphasised the need for informed choice and provision of care within communities.

*“I think about what midwifery is and how to define it. But one thing that is sort of professionally, really integral to the work is choice. And so that right away is the commitment to informed choice, a commitment to supporting a person to have autonomy within the context of their communities, and that those things are not separate from one another.”* [Interview 115]

*“Health care should be provided in the community where you live, I feel really strongly about this.[…] So for me, it's making sure we meet people where they are instead of forcing them to come to where they shouldn't have to be.”* [FGD 1]

Participants also discussed how midwives are filling gaps in the health system, particularly in the health workforce.

*“Utilising midwives in this work [abortion] is beneficial for the midwifery workforce and then subsequently for the health workforce overall in terms of filling that gap in primary care, but then also, of course, for the public and patients and just like the population as a whole. Like it just has this kind of multipronged midwives providing more sexual and reproductive health and broadening that scope of practice has benefits in all these areas on all these levels.”* [Interview 114]

Participants also shared how supporting midwives to provide comprehensive sexual and reproductive healthcare helps to address burnout within the profession and support sustainability. Burnout and attrition are serious threats to the health workforce and sustainability of midwifery. Participants discussed that the profession's sustainability requires broadening the currently narrow application of midwifery care in Canada beyond pregnancy, birth and the first 6–8 weeks of the postpartum and newborn period. Providing alternatives to demanding midwifery call schedules related to attending births, was viewed as an enabler of midwifery sustainability.

*“What can midwives do beyond three months postpartum? What can midwives do in terms of health promotion and clinical care for well-person visits that are not with people who are pregnant? Like what are midwives’ goals in the health care system? I think is a big question. And so that's why we need to look at scope of practice because midwives can do so much more, and we're not letting them.”* [Interview 115]

*“I think it will be a great opportunity for those midwives that are retiring. And for those of us who don't want to work in this model of care, and you can continue to keep your license and your skills. Midwives are waiting and resigning from the positions because there's no other options for them to work. And let's be clear that I think many people know that. But abortion care is not only about taking care of the public, but it's also taking care of the health system that is already not working well.”* [Interview 103]

*“But those midwives I was talking about who like can't really practice, kind of like retiring out or they're taking their retirement for midwifery care because they can't stay up all the time at night. But they're still, you know, brilliant clinicians, and maybe they want to work two days a week. You can mix them in.”* [Interview 102]

## Discussion

Access to abortion in Canada varies significantly and greatly depends on an individual’s geographical location, financial resources, and whether they have access to a primary care health professional.^2^ Eighteen percent of people who can get pregnant live over 100 km away from an abortion clinic, and the rise of crisis pregnancy centres may pose additional barriers to care through mis- and disinformation and delaying access to services.^2^ Our findings highlight that midwives are a vital element of the solution. Midwives are ideal abortion care providers, as their approach and midwifery philosophy align with supporting equitable access to care. Our research is the first of its kind in Canada to qualitatively: (1) learn from midwives’ experiences about the current regulatory barriers that need to be addressed, (2) explore how midwives are currently navigating health system barriers to provide abortion care, and (3) develop evidence-informed solutions for the integration of midwife-led abortion care into practice based on midwives’ values, needs, and preferences.

Our findings align with previous research supporting that midwives in Canada are interested and want to include abortion care in their scope of practice.^34^ Abortion care is a part of the care that midwives provide and part of their role as health professionals.^35^ We also know from previous research that midwives are clinically very well suited to the provision of abortion care.^36–38^ With respect to access to and experiences with abortion services among Indigenous women and 2SLGTBQIA+ people in Canada, midwives play an especially important role in supporting culturally safe comprehensive sexual and reproductive health, including abortion care.^8^ Essential investments and innovations are needed in Indigenous midwifery and the restoration of community-based midwifery services rooted in Indigenous knowledge and values.^8^

### National Strategy for Midwife-led Abortion Care

As part of the broader CART Access Project, which this study falls under, the Canadian Association of Midwives created a *National Strategy for Midwife-led Abortion Care*.^39^ Our research findings informed the development of the national strategy, and it is the first time a midwifery association has developed an evidence-informed tool to guide midwives, other health professionals, and health system decision-makers. The strategy presents the main components of the midwifery model of abortion care (see Appendix A), along with five key recommendations to guide the next steps for implementing midwife-led abortion care in Canada.

The midwifery model of abortion care centres on a holistic lifespan approach to midwife-led abortion care, which recognises that sexual and reproductive health needs span the life course and that midwives share knowledge in ways that support bodily autonomy. The model outlines the ten core components of an enabling environment that, when combined, create conditions for midwives to provide abortion care in health systems across Canada and within First Nations, Inuit, and Métis communities. The model then considers four key implementation questions: (1) by whom abortion care is delivered, (2) what type of abortion care is delivered, (3) where abortion care is delivered, and (4) how abortion care is delivered.

Based on the evidence generated in the CART Access project, we co-created five action-oriented next steps for the integration of midwives as abortion care providers across health systems in Canada and within First Nations, Inuit, and Métis communities.

1. Provide comprehensive care, which means health systems prioritising and making the necessary investments into comprehensive sexual and reproductive care, including better integration of midwives into health systems.
2. Support midwives’ associations, as they are the professional bodies that represent midwifery and are critical change agents.
3. Optimise midwives’ scope of practice to harness the full potential of midwives in abortion care by recognising and leveraging midwives’ unique skill sets and supporting midwives to provide abortion care services independently.
4. Strengthen prescribing authority to ensure midwives can provide timely and effective abortion care, which includes removing current provincial and territorial restrictions on midwives’ prescribing authority.
5. Establish payment mechanisms that are fair, sustainable, and inclusive of abortion care services.

### Limitations

There were three main limitations to the research. The first limitation was the challenges of engagement in the project. Specifically, as part of the project, we focused on the critical voices in midwifery and abortion care. While we were able to include racialised midwives, 2SLGBTQI+ midwives, midwives with disabilities, midwives who are immigrants to Canada, and internationally educated midwives in the project, our engagement with Indigenous midwives was not as comprehensive as we would have liked. The project funding cycle was limited to one year, posing a barrier to relationship building, which takes time. While the Canadian Association of Midwives works in partnership with the National Council of Indigenous Midwives, which is the voice of Indigenous midwifery, it took time to build meaningful relationships specific to this project. There are deep legacies of historical and continued racism that Indigenous peoples in Canada face, which include forced and coerced abortions and sterilisation. There were initial reservations about the project and a need for time for us as a project team to be able to show our intent and approach to the topic area. The National Council of Indigenous Midwives is now a formal project steering committee member.

The second limitation we recognise is concerning national-level work in Canada. Healthcare in Canada is primarily organised and delivered at the provincial/territorial level. There is also federally organised healthcare and combinations of federal and provincial/territorial healthcare as set out by historical and modern agreements (e.g. the Indian Act, the Canada Health Act, the Canada Health Transfer, and the Constitution Act). As such, the implementation of midwifery varies by province, territory, and sometimes by region. This means that how midwifery care is organised and delivered can vary greatly depending on where a person lives.

The final limitation is related to the participant population. Our inclusion criteria focused on midwives who were either currently providing abortion care or in the process of implementing abortion services. As expected, this focus shaped the sample and resulted in a participant group generally supportive of midwives providing abortion care. While this enabled an in-depth understanding of the facilitators and challenges from the perspective of those actively involved in abortion care, it did not capture the full spectrum of views, including those who may experience moral distress. However, participants did speak about the presence and influence of anti-choice healthcare providers, including barriers within midwifery education and practice settings, as well as broader social and political influences. These perspectives are reflected in the theme of barriers to the integration of midwife-led abortion care.

## Conclusion

Many barriers to culturally safe, appropriate, and timely access to abortion care persist in Canada. Midwives possess the skills, knowledge, and competencies required to provide abortion care and are a crucial component of a broader movement committed to achieving reproductive justice. With the removal of unnecessary provincial and territorial regulatory restrictions and barriers, midwives can increase their contributions to sexual and reproductive care and further enhance access for all.
